# FINDINGS FROM NSHAP: SOCIAL CONNECTEDNESS, HEALTH INDICATORS, MEDICATION EFFECTS, AND PREDICTING MORTALITY

**DOI:** 10.1093/geroni/igad104.1557

**Published:** 2023-12-21

**Authors:** Lissette Piedra, Amelia Karraker

## Abstract

The National Social Life, Health, and Aging Project’s broad range of both social measures, and objective and self-reported health measures enable detailed analysis of the intersections between these fundamental aspects of older adults’ lives. The papers in this symposium explore various aspects of these topics from different angles. The first explores employment as an important form of social participation, establishing that full-time employment among respondents is associated with better cognitive function, and less ADL and IADL difficulties. The second examines how social isolation affects men and women differently. Social Networks are the focus of the third paper and compare family to friendship ties. Using NSHAP’s unique medication log, Wilder examines sleep disturbances and the prevalence of respondents taking medications with somnolence as an adverse event, demonstrating the need for more research into how this might affect older adults’ health and well-being. Li uses NSHAP data to develop machine learning models to predict 10-year mortality of older adults in the US which perform with better accuracy than logistic regression.

